# RNA virus receptor Rig-I monitors gut microbiota and inhibits colitis-associated colorectal cancer

**DOI:** 10.1186/s13046-016-0471-3

**Published:** 2017-01-05

**Authors:** Houbao Zhu, Wang-Yang Xu, Zhiqiang Hu, Hongxin Zhang, Yan Shen, Shunyuan Lu, Chaochun Wei, Zhu-Gang Wang

**Affiliations:** 1 State Key Laboratory of Medical Genomics, Research Center for Experimental Medicine of Rui-Jin Hospital, Shanghai Jiao Tong University School of Medicine, Shanghai, China; 2 Model Organism Division, E-Institutes of Shanghai Universities, Shanghai, China; 3 School of Life Sciences and Biotechnology, Shanghai Jiao Tong University, Shanghai, China; 4 Shanghai Research Center for Model Organisms, Shanghai, China; 5 Shanghai Center for Bioinformation Technology, Shanghai, China; 6 Research Center for Experimental Medicine, Rui-Jin Hospital, 197 Ruijin Road II, Shanghai, 200025 China

**Keywords:** Rig-I, Gut microbiota, Colorectal cancer, Mouse model, High-throughput sequencing

## Abstract

**Background:**

Retinoic acid-inducible gene-I (Rig-I) is an intracellular viral RNA receptor, which specifically recognizes double-stranded viral RNA initiating antiviral innate immunity. Increasing evidences showed that Rig-I had broader roles in antibacterial immunity and cancer protection. However, the potential roles and mechanisms of Rig-I in gut flora regulation and colorectal cancer (CRC) progression remain unclear.

**Methods:**

Immunohistochemistry was performed to detect Rig-I protein in 38 pairs of CRC tissue and matched adjacent mucosa, and immunofluorescence and western blot were also used to detect Rig-I protein expression in AOM/DSS-induced mice CRC samples. High-throughput sequencing was conducted to evaluate gut microbiota changes in *Rig-I*-deficient mice. Immunofluorescence and flow cytometry were used to detect IgA expression. Additionally, real-time quantitative PCR was performed to detect RNA expression in mouse intestines and cultured cells, and western blot was used to detect phosphorylation of STAT3 in IL-6-stimulated B cell line.

**Results:**

Rig-I was downregulated in human and mouse CRC samples and *Rig-I*-deficient mice were more susceptible to AOM/DSS-induced colitis-associated colorectal cancer (CAC). Furthermore, *Rig-I*-deficient mice displayed gut microbiota disturbance compared to wild type mice. IgA, Reg3γ and Pdcd1 levels were decreased in intestines of *Rig-I*-deficient mice. Phosphorylation of STAT3 in IL-6-stimulated 1B4B6 was decreased.

**Conclusion:**

Rig-I could regulate gut microbiota through regulating IgA and IL6-STAT3-dependent Reg3γ expression. Besides, Rig-I could inhibit CRC progression.

**Electronic supplementary material:**

The online version of this article (doi:10.1186/s13046-016-0471-3) contains supplementary material, which is available to authorized users.

## Background

CRC is one of the leading causes of death among cancer patients worldwide [1]. Recent studies have demonstrated that infection and chronic inflammation promote the development and progression of a variety of cancers in humans [2, 3], including CAC [4, 5]. A frequently used mouse model, chemically induced by administration of azoxymethane (AOM) plus dextran sodium sulfate (DSS) drinking water, successfully simulates the whole process that inflammatory bowel disease (IBD) induces CRC [6–8]. Using this model, researchers have highlighted the critical roles of the transcription factors NF-κB and STAT3 in colorectal cancer progression [9–12].

The gut microbiota is a complicated population consisting of countless microbes that reside within the gastrointestinal tract and form a complex ‘super-organism’ with their host [13]. Defect in the regulation of bacterial sensing and homeostasis, or alteration in the composition of the gut microbiota may disturb the symbiotic relationship between microbes and hosts, which may further promote the development of IBD and CRC [13, 14]. Increasing evidences demonstrate the crucial role of the gut microbiota in carcinogenesis [15–17], and several bacterial groups have been identified as CRC-associated bacteria, including *Bacteroidetes*, *Proteobacteria* and *Fusobacterium* [13, 18, 19]. Host recognition of various microbial components known as pathogen-associated molecular patterns (PAMPs) mainly depends on pattern-recognition receptors (PRRs), such as Toll-like receptors (TLR), Nod-like receptors (NLRs), Rig-I-like receptors (RLRs) and C-type lectin receptors (CLRs) [20–22].

Retinoic acid-inducible gene-I (Rig-I, also known as DDX58), a main member of RLRs, is an intracellular viral RNA receptor, which specifically recognizes double-stranded viral RNA initiating antiviral innate immunity [23]. In our previous studies, we found that Rig-I knock-out (*Rig-I* ^−/−^) mice were susceptible to colitis, which was similar with human IBD, accompanied with decreased expression of G protein subunit α-i2 (Gαi-2) and abnormal T cell activation [24]. *Rig-I* ^−/−^ mice were also found to be more susceptible to infection with E. coli as compared to wt mice due to decreased phagocytosis of bacteria [25]. Furthermore, *Rig-I* ^−/−^ mice were susceptible to spontaneous infection with a commensal bacterium, *S. xylosus*, in the skin around eyes and neck, accompanied with defects in B cell development, specific IgG3 immunoglobulin class switch recombination (unpublished data) and p105 translational inhibition [26]. These results indicate that the regulatory functions of Rig-I are strikingly broad. It plays a crucial role not only in antiviral responses but also in antibacterial responses. Additionally, Rig-I was down-regulated in intestinal epithelial compartment of IBD patients, accompanied with apparent disorder of intestinal flora [27]. And 18% of IBD patients may eventually develop into colorectal cancer [27]. But the influence of Rig-I on the colorectal carcinogenesis and its association with gut microbiota are still unclear.

In the present study, we analyzed the composition of gut microbiota in *Rig-I* ^−/−^ mice and evaluated its effect on colorectal carcinogenesis. We found that Rig-I had an important role in suppressing the development of CRC and in the regulation of intestinal flora. This provides novel insights on CRC and reveals the potential therapeutic strategy.

## Methods

### Mice and tumor induction

The Rig-I-deficient (*Rig-I* ^−/−^) mice had been reported previously [24]. Mice were kept in specific-pathogen free conditions and fed by free access to a standard diet and water. All experiments were approved by the Animal Ethics Committee of Rui-Jin Hospital, Shanghai Jiao Tong University School of Medicine. The induction of tumors had been described previously [6–8]. 8-10-week-old sex-matched wild type and *Rig-I* ^−/−^ mice were injected with 10 mg/kg AOM (Sigma) intraperitoneally. After 1 week, mice were given with 2.5% DSS (35,000–50,000 kDa, MP Biomedicals) in drinking water, then followed with regular water for 2 weeks. This cycle was repeated thrice. At the end of the experiment, all the mice were sacrificed and the colon samples were collected, measured and fixed for subsequent paraffin embedding and histological analysis. Macroscopic colon tumors were counted and their diameters were measured with the caliper. Tumor load was calculated as the sum of all tumors’ diameters [6].

### Tissue microarrays of RIG-I expression

Specimens from 40 primary colorectal adenocarcinoma patients who underwent surgery at the Shanghai Rui-Jin Hospital were made into formalin-fixed, paraffin-embedded serial sections (4-μm thick and 1-mm wide cylinders) of tissue microarrays. Samples from the same patient consisted of 1 adjacent and 2 tumor tissues. Written informed consent was obtained from all involved patients. The experimental protocol was approved by the Ethics Committee of Rui-Jin Hospital, and the research was carried out according to the provisions in the Declaration of Helsinki. Detailed Rig-I staining was performed according to the standard immunohistochemical analysis protocol described in Immunofluorescent and immunohistochemical analysis section, and the staining intensity was scored by pathologist in a double-blind fashion as ‘−’, negative; ‘+’, weakly stained; ‘++’, moderately positive and ‘+++’, strongly positive. The standard graphs of scoring were shown in Additional file 1: Figure S1.

### Histological scoring

All the scoring procedures were performed by pathologists in a double-blind fashion. Mucosal inflammation was scored according to previous description [8, 24]: 0, normal morphology; 1, focal inflammatory cell infiltrate around the crypt base; 2, diffuse infiltration of inflammatory cells around the crypts or erosion/destruction of the lower one-third of the glands; 3, erosion/destruction of the lower two-thirds of the glands or loss of all the glands but with the surface epithelium remaining; and 4, loss of all the glands and epithelium. Dysplasia was scored as follows [8, 15]: 0, no dysplasia; 1, mild dysplasia characterized by aberrant crypt foci (ACF), +.5 for small gastrointestinal neoplasia (GIN) or multiple ACF; 2, moderate dysplasia with GIN, +.5 for multiple occurrences or small adenoma; 3, severe or high grade dysplasia restricted to the mucosa, +.5 for adenocarcinoma, invasion through the muscularis mucosa; 4, adenocarcinoma, full invasion through the submucosa and into or through the muscularis propria.

### Commensal depletion

Mice were initially given a treatment of 0.5 mg/ml vancomycin, 1 mg/ml of neomycin, 1 mg/ml ampicillin and 1 mg/ml of metronidazole for 4 weeks. Freshly prepared antibiotics were supplied every week. Then, drinking water was further supplemented with 170 μg/ml of gentamicin, 125 μg/ml of ciprofloxacin, 1 mg/ml of streptomycin and 1 mg/ml of bacitracin as previously described [28, 29]. Microbiota of the small intestine was determined after plating the diluted samples on universal and differential media for aerobes and anaerobes. After mice were treated with the antibiotics for 5 weeks, colon cancer was induced in wt or *Rig-I* ^−/−^ mice as described above.

### Sample collection, sequencing and bioinformatics analysis for intestinal microbiota

To obtain well-matched wt and *Rig-I* ^−/−^ mice for the comparison of intestinal microbiota, wt and *Rig-I* ^−/−^ littermates from a single heterozygous intercross were selected to start homozygous intercross that were housed separately from this point on. Faeces samples were collected immediately after defaecation at different ages (2, 4, 8, 12 and 16 weeks old) and stored at −80 °C. Bacterial DNA was extracted using QIAamp DNA Stool Mini Kit (QIAGEN) according to the manufacturer’s instructions and used as template in the amplification of the V3 region of 16S rRNA gene. The forward primer was 5′-NNNNNNNNTACGGGAGGCAGCAG-3′, and the reverse primer was 5′-NNNNNNNNATTACCGCGGCTGCTGG-3′, where the underlined sequence was the universal bacterial primer, and the ‘NNNNNNNN’ was the unique eight-base barcode used to distinguish PCR product from different samples. PCR reaction conditions were as follows: each 25 μl PCR reaction mixture contained 0.125 μl of ExTaq polymerase (TAKARA), 2.5 μl of the corresponding 10× ExTaq amplification buffer, 0.125 μl of 0.1% BSA, 2 μl of dNTP, and 1 μl of each primer (10 pmol), and 25 ng of total faecal DNA. PCR reactions were run in a thermocycler PCR system using the following program: 3 min denaturing at 94 °C followed by 20 cycles of 1 min at 94 °C (denaturing), 1 min for annealing (1 °C reduced for every 2 cycles from 65–57 °C followed by 1 cycle at 56 °C and 1 cycle at 55 °C), and 1 min at 72 °C (elongation), with a final extension at 72 °C for 6 min. Sequencing was performed at the Chinese National Human Genome Center at Shanghai using 454 GX-FLX (Roche). The resulting sequences were available in the SRA database under BioSample accessions SAMN05756210-SAMN05756289. Sequencing data were analyzed with Mothur v1.32.0 [30] and the detailed procedures were shown in Additional file 1: Figure S2. Totally, 180,798 raw sequences were obtained. Sequences were qualified and linkers and primers were removed (2 mismatches were allowed). Sequences were then divided according to the barcodes (no mismatch was allowed) and 130,891 sequences remained. 31,644 unique sequences were selected to simplify the computation. These sequences were aligned to SILVA-compatible alignment database (SILVA 115 release) [31] and sequences out of V3 regions were further removed. Unique sequences aligned to the same reference sequence were merged to a new unique sequence. At this time, 6994 unique sequences (130,862 total sequences) remained. 916 chimeras sequences (110 unique sequences) were removed by using these sequences as their own reference. Contaminants including chloroplasts and mitochondria were also removed based on RDP (version 9) [32] annotation and 129,832 sequences (6877 unique sequences) remained. Next, sequences were clustered to 2346 OTUs at species level (cutoff = 0.03). An extra process was carried out to remove OTUs with very low abundance in all samples, which were likely to be spurious. Chi-square tests based on binomial distribution were introduced to remove OTUs with abundance less than 1% (*P*-value < 0.05). 379 OTUs with 108,241 sequences were obtained. The taxonomy information of the OTUs was inferred through RDP and GREENGENES (May, 2013) [33] databases. The phylogenetic tree was built with Neighbor-Joining methods with 1000 bootstraps. Parsimony method (aka P-test) was used to infer the differences of species compositions of each group. Chao index and inverse Simpson diversity index were calculated.

### Western blot and antibodies

Tissue or cell lysates were prepared in lysis buffer (1% Nonidet P-40, 0.5% sodium deoxycholate, 0.1% SDS in PBS) with freshly supplemented protease inhibitors cocktail (Roche). Proteins were separated by SDS-PAGE and transferred to nitrocellulose membranes. The individual proteins were detected using the indicated antibodies. All antibodies used in this program were as follows: anti-RIG-I (Santa Cruz), anti-RIG-I (Abcam), anti-PD1 (Thermo Scientific), anti-B220 APC (eBioscience), anti-IgA (eBioscience), anti-Akt (Cell Signaling), anti-pAkt (Ser473; Cell Signaling), anti-STAT3 (Cell Signaling), anti-pSTAT3 (Cell Signaling), anti-ERK1/2 (Cell Signaling), anti-pERK1/2 (Cell Signaling), anti-p38 (Cell Signaling), anti-p-p38 (Cell Signaling), anti-p50 (Neomarker), anti-p65 (Neomarker), anti-RelB (Santa Cruz), anti-p27 (Santa Cruz), anti-Gαi2 (Santa Cruz) and anti-PCNA (Santa Cruz).

### RT-qPCR

The RT-qPCR was performed according to the MIQE guidelines [34]. Total RNA was isolated from tissues or cells using TriPure reagent (Roche) according to the manufacturer’s instructions, and then was reverse-transcribed into cDNA with RT regent kit (Takara). The gDNA erasing reaction conditions were as follows: each 10 μl reaction mixture contained 2 μl of gDNA Eraser Buffer, 1 μl of gDNA Eraser and 1 μg RNA. The mixtures were incubated for 2 min at 42 °C. The reverse-transcribed reaction conditions were as follows: each 20 μl PCR reaction mixture contained 1 μl of Enzyme Mix, 1 μl of Primer Mix, 4 μl of PrimeScript Buffer and 10 μl of reaction mixture above. PCR reactions were run in a thermocycler PCR system using the following program: 15 min at 37 °C followed by 5 s at 85 °C. Quantitative PCR was carried out using SYBR Green PCR kit (Takara) according to the manufacturer’s instructions. In brief, each 20 μl PCR reaction mixture contained 10 μl of SYBR Premix Ex Taq, 0.8 μl of Primer Mix (5 μM) and 1 μl of cDNA solution. Amplifications were performed in the Mastercycler ep realplex machine (Eppendorf) using the following program: 30 s denaturing at 95 °C followed by 40 cycles of 5 s at 95 °C and 30 s at 60 °C, with a final stage of melting curve. Relative transcript quantities were calculated using the ΔΔC_q_ (quantification cycle) method with β-Actin as the endogenous reference gene. Each sample was analyzed in triplicate and the experiment was replicated three times. Primer sequences were designed using DNAMAN or accessed from PrimerBank [35], and the sequences were listed in Additional file 1: Table S1.

### Isolation of PP cells for flow cytometry

Peyer’s patches (PPs) were collected from wt and *Rig-I* ^−/−^ mice and were washed three times in cold PBS. Cell suspensions were obtained by passing tissues through a 200-μm nylon mesh. The PP lymphocytes were stained with indicated antibodies for 30 min at 4 °C. The analysis was performed on FACScalibur (Becton Dickinson) and the data obtained were processed with the FlowJo software.

### Immunofluorescent and immunohistochemical analysis

Paraffin-embedded slides were deparaffinized. Antigen unmasking was carried out by incubation in 92–98 °C water bath in 10 mM sodium citrate buffer for 30 min. Slides were incubated with indicated primary antibodies in PBS containing 1% BSA and 10% goat serum overnight at 4 °C. For immunohistochemistry, biotinylated secondary antibodies were added and incubated at room temperature for 1 h. Then, streptavidin-HRP was added and after 40 min the sections were stained with DAB substrate and counterstained with hematoxylin. For immunofluorescence, the slides were incubated with fluorescent secondary antibodies and counterstained with DAPI.

### Cell culture and knock down

Mouse B cell line 1B4B6 [36] was a kind gift from Dr. Amy. L. Kenter and *Rig-I* knock down was described previously [26]. In brief, the 1B4B6 cell line was cultured in RPMI 1640-based complete medium containing 10% FBS and was infected with retrovirus vector expressing *Rig-I* siRNA and EGFP. FACS-sorted EGFP^+^ cells were further selected with puromycin (8 μg/mL) for 1 week. Subsequently, single **Rig-I**–RNAi and control cell clones were expanded for further study.

### Statistical analysis

Quantitative data were presented as mean ± SD unless otherwise specified, and comparisons between two groups were analyzed using two-tailed Student’s t-test. Distribution of Rig-I expression analysis was compared using Chi-square test. Survival analysis was performed using the Kaplan-Meier method and compared using log-rank test. *P* < 0.05 was considered statistically significant.

## Results

### Rig-I expression was decreased in CRC

**Fig. 1:**
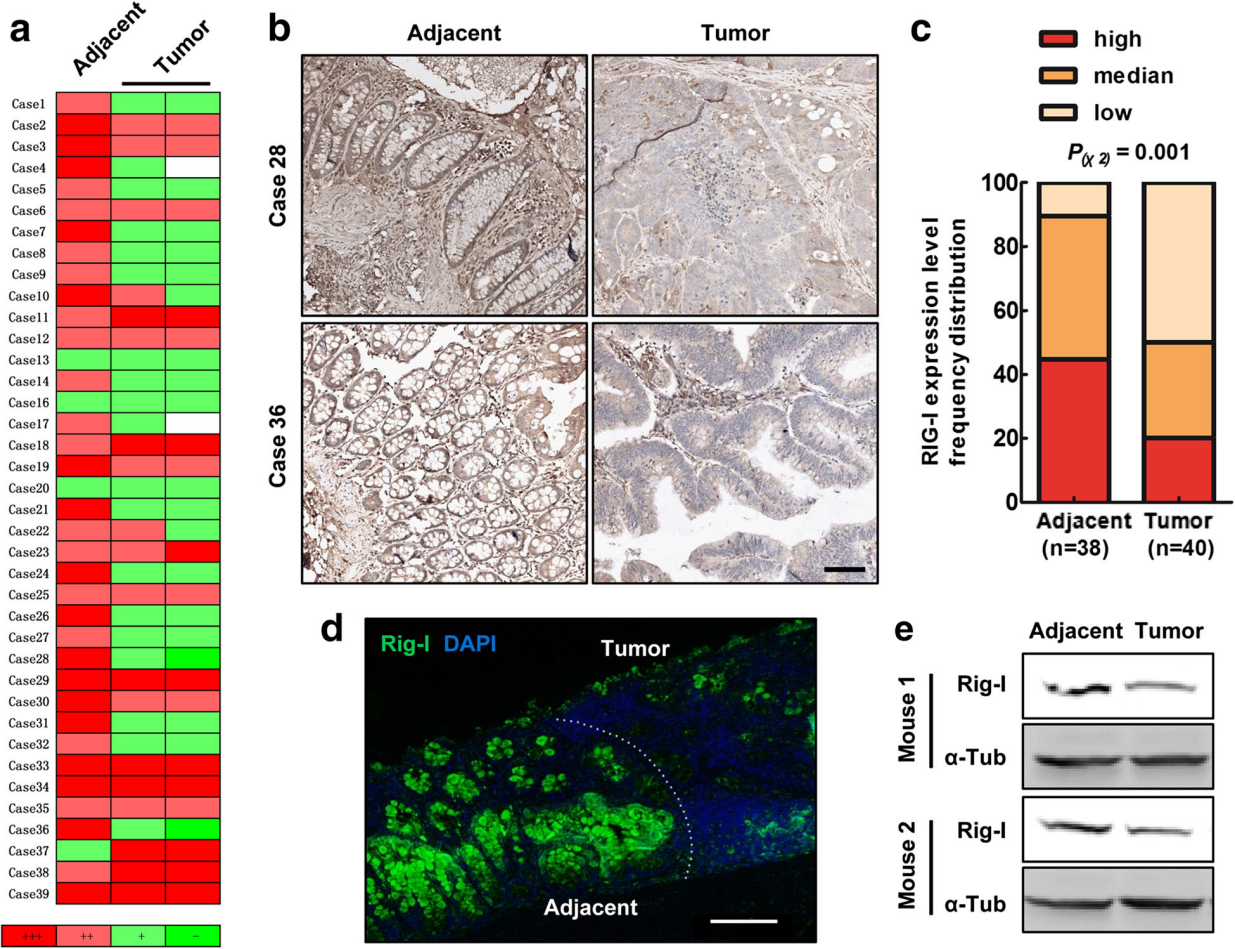
Rig-I was down-regulated in human and mouse colorectal tumors. **a** Heat map of RIG-I immunohistochemical scores in tissue microarrays containing 38 colorectal cases (one adjacent and two tumor tissues). **b** Representative RIG-I immunohistochemical staining in human colorectal tumors and adjacent tissues. **c** The score distribution of RIG-I staining in adjacent tissues and tumors. **d** Rig-I immunofluorescent staining in AOM/DSS-induced mouse colorectal tumors. **e** Immunoblot analysis of Rig-I expression in induced mouse colorectal tumors. Scale bar, 100 μm

Rig-I was reported to be associated with several cancers [37–39]. To study the role of Rig-I in colorectal carcinogenesis, we collected 38 samples of CAC cases to detect Rig-I protein expression using immunochemistry method. We found that Rig-I expression was decreased in tumor sites of the majority of the cases (Fig. 1a). Figure 1b showed the presented views of stained sections. Besides, distributions of Rig-I expression were significantly different between tumor and adjacent tissues (Chi-square test, *P* = 0.001) (Fig. 1c). Furthermore, we also found that Rig-I expression was decreased in AOM/DSS-induced CAC samples through both immunofluorescence (Fig. 1d) and Western blot analysis (Fig. 1e). All the above results demonstrated that Rig-I expression was decreased in CRC samples.

### *Rig-I*^−/−^ mice were more susceptible to induced CAC

**Fig. 2:**
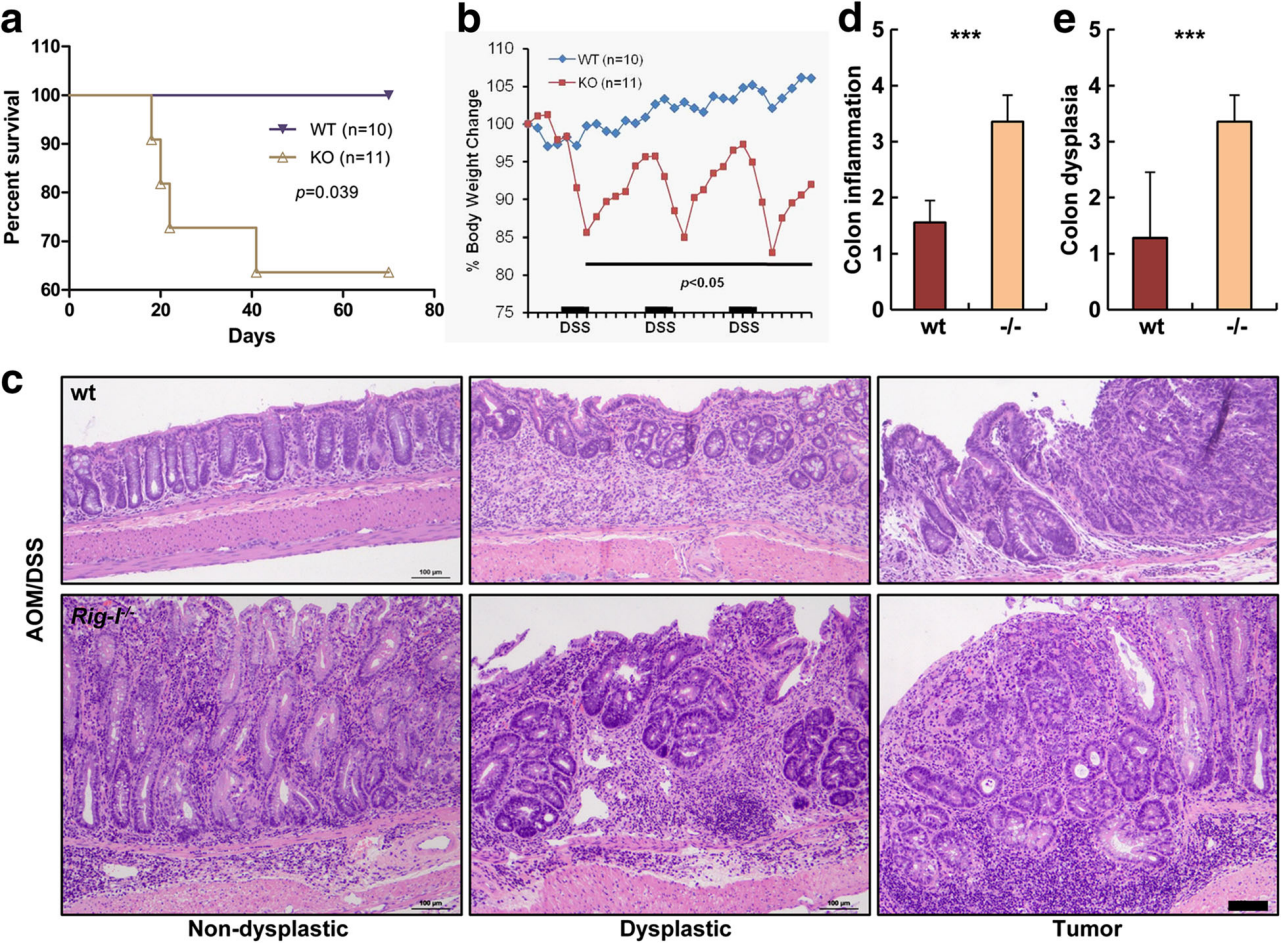
*Rig-I* ^−/−^ mice were susceptible to induced colorectal tumors. **a** Survival curve of AOM/DSS-treated wt and *Rig-I* ^−/−^ mice during the study period. **b** Alteration of body weight during the study period. The values were expressed as a percentage of body weight on day 0. **c** Tumors in the AOM/DSS-induced wt and *Rig-I* ^−/−^ CAC model. **d** Colitis severity scores. **e** Dysplastic scores. Scale bar, 100 μm

Though Rig-I expression was down-regulated in most CRC samples, it was still unclear whether Rig-I deficiency could promote CAC development and progression in vivo. To answer this question, we induced wt and *Rig-I* ^−/−^ mice to develop CAC using AOM plus DSS under the same conditions. The induction procedure was shown in Additional file 1: Figure S3a. During the experimental procedure, *Rig-I* ^−/−^ mice showed reduced survival time and higher mortality than wt mice (Fig. 2a, *P* = 0.039). Additionally, *Rig-I* ^−/−^ mice showed obvious weight loss (Fig. 2b), loose stools and bloody stools (data not shown) compared with wt mice. Moreover we found that the colons of *Rig-I* ^−/−^ mice were much shorter and thicker than those of wt mice (Additional file 1: Figure S3b). This indicated that *Rig-I* ^−/−^ mice underwent more serious induced colitis, which was also confirmed by pathological staining and scoring (Fig. 2c and d). As is known, colitis is a critical promoter of CAC progression. Here, we found a striking difference between *Rig-I* ^−/−^ and wt mice with respect to development of dysplasia and tumors. The untreated colons were comparable between *Rig-I* ^−/−^ and wt mice (Additional file 1: Figure S3c), but *Rig-I* ^−/−^ mice loaded more severe dysplasia and adenocarcinoma (Fig. 2c) and showed higher histological dysplastic scores (Fig. 2e). The number of cases for each given score related to Fig. 2d and e was shown in Additional file 1: Table S2. Taken together, these results showed that Rig-I loss could promote the progression of induced CAC in mice.

### *Rig-I*^−/−^ mice displayed intestinal flora disturbance

**Fig. 3:**
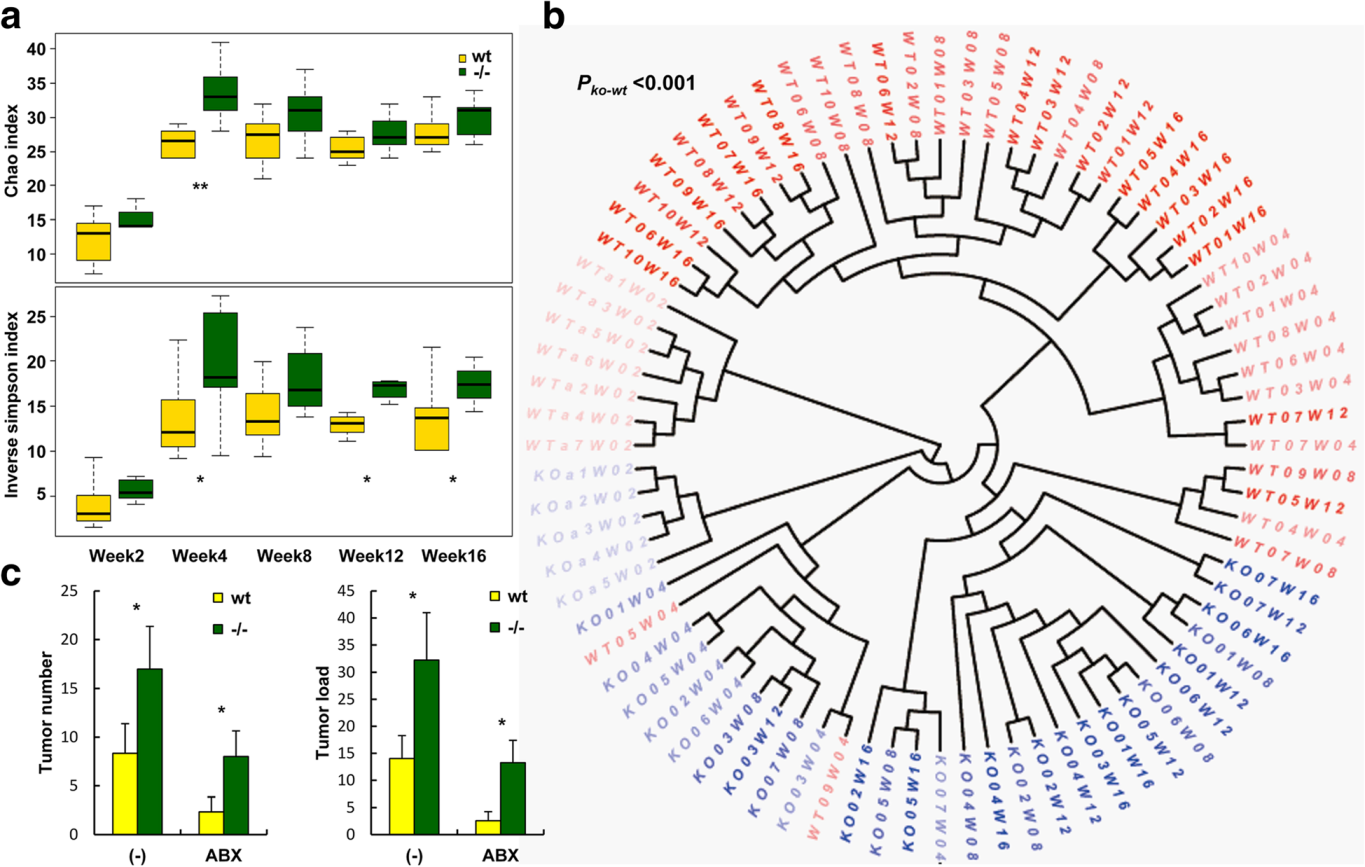
*Rig-I* ^−/−^ mice displayed gut microbiota disturbance. **a** Chao index and inverse Simpson diversity index were shown for each genotyped group at five age points. Data were shown as means ± SEM. **b** Neighbor-Joining phylogenetic tree of bacterial communities. The different color depths represented samples of different age points. In the sample ID, the first two letters “WT” and “KO” represented corresponding wild-type and *Rig-I* ^−/−^genotypes, respectively, and the numbers close to the letters represented different individuals, and the last letter “W” and numbers represented corresponding weeks. **c** Tumor number and tumor load in antibiotics-treated mice

Intestinal flora has a key role in colorectal carcinogenesis [13]. To evaluate the effect of Rig-I on intestinal microbiota regulation, we collected stool samples from *Rig-I* ^−/−^ and wt mice in different ages. Then, we sequenced and studied the amplicons of bacterial 16S rRNA V3 regions using 454 pyro-sequencing method. As a result, we found that the gut microbiota in *Rig-I* ^−/−^ mice showed more species richness and diversity generally (Fig. 3a, *P*_(Chao index)_ = 1.39 × 10^−4^; *P*_(Inverse simpson index)_ = 8.74 × 10^−6^) than wt mice. Besides, the phylogenetic analysis of the bacterial communities suggested that the gut microbiota in *Rig-I* ^−/−^ and wt mice differed clearly (Fig. 3b, *P* < 0.001). Moreover, combined with the *P* test [30] on the Neighbor-Joining tree (Additional file 1: Figure S4a), we found that gut microbiota of both *Rig-I* ^−/−^ and wt mice was in flux from birth. Principal component analysis also showed that the gut microbiota of *Rig-I* ^−/−^ and wt mice were different (Additional file 1: Figure S4b).

Furthermore, to determine whether the susceptibility of *Rig-I* ^−/−^ mice to induced CAC was caused by imbalanced gut flora, we removed most of the bacteria via treating mice with a cocktail of antibiotics (Abx). Interestingly, *Rig-I* ^−/−^ mice were still more susceptible to induced CAC than wt mice although all mice held decrease tumor number and tumor load than that before Abx intake (Fig. 3c). This suggested that gut flora disturbance was not the fundamental cause of the susceptibility of *Rig-I* ^−/−^ mice to induced CAC. This indicated that the different susceptibility might result from Rig-I-mediated signaling pathway. However, when we tried to detect the signaling changes in *Rig-I* ^−/−^ mice, we did not found a crucial molecule, activation state of which contributed to CAC development and progression, in untreated samples (Additional file 1: Figure S5a) or in AOM/DSS-induced tumorigenic samples (Additional file 1: Figure S5b).

### Rig-I deficiency led to decreased intestinal IgA level

**Fig. 4:**
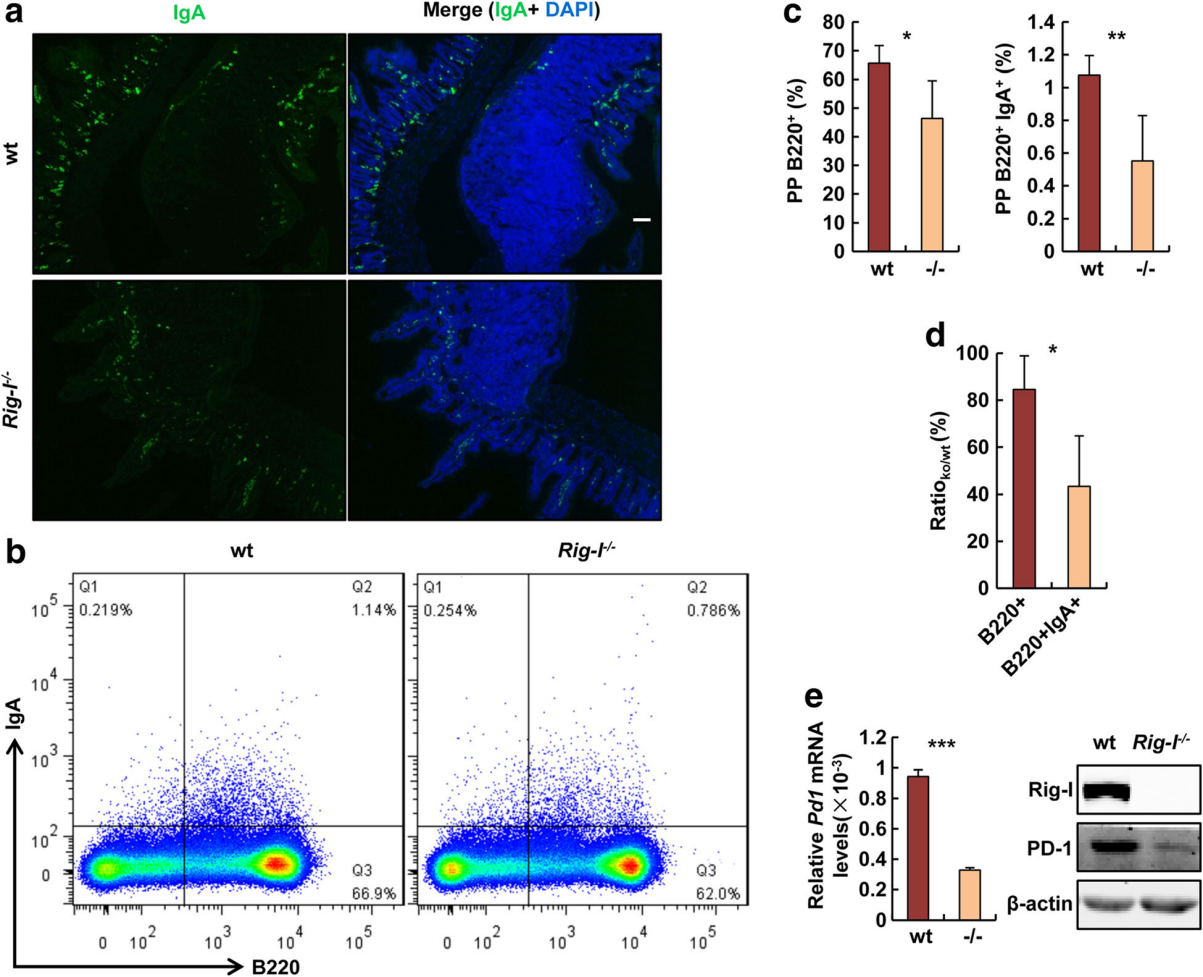
IgA was decreased in *Rig-I* ^−/−^ intestines. **a** IgA staining of intestinal sections. **b** Flow cytometric analyses of IgA cells in PPs of wt and *Rig-I* ^−/−^ mice. Numbers indicated the percentages of cells in the marked gates. **c** Graphs showed percentages of the indicated cells. **d** Ratio of the indicated cells between *Rig-I* ^−/−^ and wt mice. **e** Pd1 mRNA and protein expression in PPs. Scale bar, 200 μm

IgA secreted by intestinal mucosa is crucial for maintaining immunological homeostasis between intestinal bacteria and mucosa [40, 41]. We found that IgA was decreased in *Rig-I* ^−/−^ PPs through immunofluorescence (Fig. 4a). We further found that IgA^+^B220^+^ cell number was decreased in PPs via flow cytometry (Fig. 4b and c). However the B220^+^ cell number was also decreased in *Rig-I* ^−/−^ PPs (Fig. 4b and c). To exclude the possibility that IgA^+^B220^+^ cell decrease was caused by total B220^+^ decrease in *Rig-I* ^−/−^ PPs, we corrected the value as a ratio of ko/wt. We found that the extent of IgA^+^B220^+^ cell decrease was larger than that of B220^+^ cell decrease (Fig. 4d). This revealed that IgA secreted by B lymphocytes was decreased in *Rig-I* ^−/−^ PPs. It was reported that Pd1 (programmed cell death 1) was a receptor, which regulated intestinal microbiota through modulating selection of IgA plasma cell repertoires [42]. In our study, we found that Pd1 expression was decreased at both mRNA and protein levels in *Rig-I* ^−/−^ PPs (Fig. 4e).

### IL6-STAT3-dependent Reg3γ expression was decreased in *Rig-I*^−/−^ mice

**Fig. 5:**
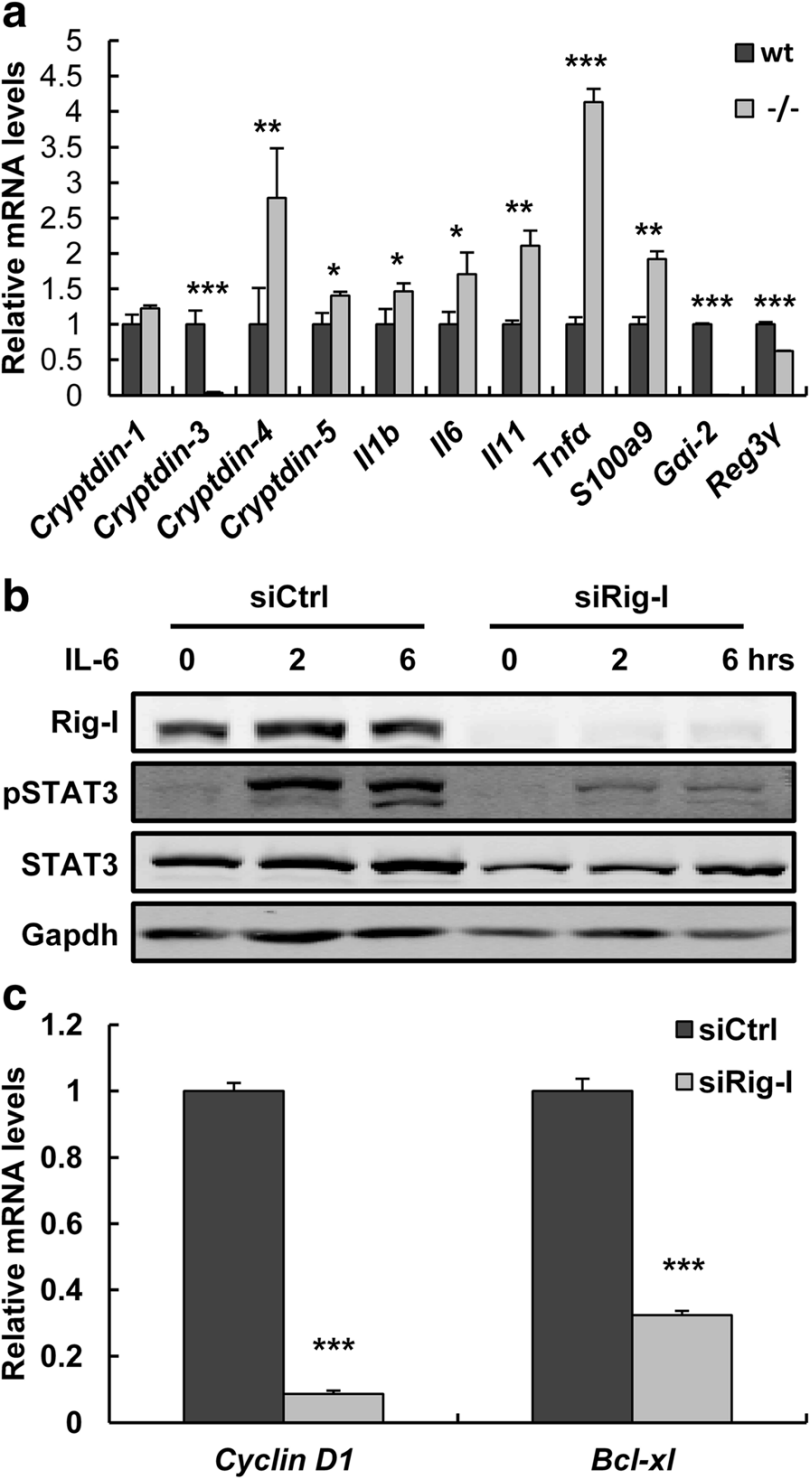
STAT3-mediated Reg3γ expression was decreased in *Rig-I* ^−/−^ mice. **a** Expression levels of antimicrobial peptides and inflammatory factors in intestines. **b** Western blot of STAT3 in Il6-treated 1B4B6 cells. **c** STAT3 target genes expression in 1B4B6 cells

Cryptdin, also known as defensin, is crucial for the balance of intestinal bacteria. It is a group of microbicidal peptides secreted by intestinal crypt Paneth cells [43]. We detected the mRNA levels of four main cryptdins in intestines via real-time qPCR. As a result, we found that compared with wt intestines, *Rig-I* ^−/−^ intestines showed increased *cryptdin-4* and *cryptdin-5* mRNA levels, decreased *cryptdin-3* mRNA levels and comparable *cryptdin-1* mRNA levels (Fig. 5a). Rig-I is a key receptor mediated antiviral immunity and it plays crucial roles in development and functional regulation of T and B lymphocytes [24], therefore we further detected the expression levels of several inflammatory factors. We found that *Il1b*, *Il6*, *Il11*, *Tnf-α* and *S100a9* were significantly increased in *Rig-I* ^−/−^ intestines (Fig. 5a). This revealed that the profile of inflammatory factors was changed extremely in *Rig-I* ^−/−^ intestines, indicating the microenvironment was changed in *Rig-I* ^−/−^ intestines. It was also reported that *Gαi2* ^−/−^ mice displayed ulcerative colitis and adenocarcinoma of the colon [44]. We found Gαi2 mRNA was decreased in *Rig-I* ^−/−^ intestines (Fig. 5a), consistent with previous result [24]. Besides, mRNA level of Reg3γ, an important regulatory factor of intestinal microbiota [45, 46], was also decreased in *Rig-I* ^−/−^ intestines (Fig. 5a). As previously reported, the transcription of Reg3γ was regulated by IL6-STAT3 [47]. We found that STAT3 was lower phosphorylated in *Rig-I* ^−/−^ intestines (Additional file 1: Figure S5a and b). Meanwhile, STAT3 was also lower phosphorylated in Rig-I-silenced mouse B cell line (1B4B6) under IL6 treatment (Fig. 5b). Consistently, cyclin D1 and Bcl-xl, two target genes of STAT3, were also down-regulated in *Rig-I*-silenced 1B4B6 (Fig. 5c). These results revealed that Rig-I regulated Reg3γ transcription through modulating the phosphorylation of STAT3.

## Discussion

Rig-I is an RNA virus receptor, which could recognize viral RNA, recruit MAVS (also known as IPS-1, VISA and Cardif) and trigger downstream of the IRF3 and NF-κB signaling pathway, inducing production of type I interferon and inflammatory factors [48–52]. We previously reported that *Rig-I* ^−/−^ mice showed decreased Icsbp1 expression and decreased STAT1 activation, thus promoting granulocytic proliferation [53, 54]. *Rig-I* ^−/−^ mice developed colitis similar to human IBD, accompanied with down-regulation of Gαi2 and disturbed T-cell homeostasis [24]. *Rig-I* ^−/−^ mice were more susceptible to infection with *E. coli* due to diminished phagocytosis of bacteria [25]. Besides, *Rig-I* ^−/−^ mice showed B cell developmental defect accompanied with block of p105 (NF-κB1) translation [26] and IgG3 class switch recombination, resulting in infection with *S. xylosus* in the skin around eyes and neck. These results suggested that Rig-I played crucial roles in regulation of antibacterial responses. Hou and colleagues [39] reported that RIG-I enhanced IFN-α response in hepatocellular carcinoma (HCC) via strengthening STAT1 activation, suggesting RIG-I is a tumor suppressor in HCC [39].

Human IBD is a disease of cellular inflammation and intestinal damage caused by multiple factors [55]. The patients with IBD show dysregulated intestinal mucosa immunity, gut flora disturbance and abnormal cytokines production. IBD susceptibility is associated with human genetic background and environment. The disturbance of gut flora is a crucial cause of suffering from IBD [55].

In the present study, we revealed that Rig-I was a crucial regulatory factor in the development of the intestinal flora in mice. Due to the changes of intestinal flora in different stages of development, we analyzed the composition of intestinal flora in 5 age points of wt and *Rig-I* ^−/−^ mice. It should be noted that the study of the microbial community in genetic modified mice was prone to systematic errors, such as the impact of cages and the genetic background of mice. We used samples of offspring of male mice from the same brood mating with another brood of female mice to avoid the effects of the feeding cage and the female mouse hormonal cycle [56]. Our study found that the variation of intestinal flora between wt and *Rig-I* ^−/−^ mice exactly existed in different ages. Our results indicated that *Rig-I* ^−/−^ mice showed obvious intestinal flora disturbance. This might be caused by Rig-I deficiency. IgA is the main immunoglobulin secreted by intestinal mucosa and is also crucial for maintaining immunological homeostasis between intestinal bacteria and mucosa [40, 41]. IgA could bind to bacteria to reduce their activities and the abilities to adhering to mucosa [57]. Unlike serum IgA isotope, secreted IgA transfers to mucosa fast and conveniently through polymeric immunoglobulin receptors as a dimer [58]. IgA and cryptdins have an important role in the regulation of intestinal flora [43, 57]. We found that IgA was decreased in PP and cryptdins were dysregulated.

About 18% of the IBD patients develop into colorectal cancer eventually all over the world. And one of the key features of IBD is gut flora disturbance. Most of CRC is associated with colitis, inflammation and chronic infection [5]. Given that *Rig-I* ^−/−^ mice showed a similar phenotype with human colitis and intestinal flora disorder, we speculated that *Rig-I* ^−/−^ mice were more susceptible to colorectal cancer. Then we confirmed this by AOM/DSS-treated mice. However, when we removed microbes in the intestines of mice using mixed antibiotics, all mice held decrease tumor number and tumor load than that before Abx intake. It is easy to understand because that the gut microbiota plays a crucial role in carcinogenesis [15–17]. But *Rig-* *I* ^−/−^ mice were still more susceptible to colorectal cancer. This suggested that Rig-I might affect colorectal cancer through a signaling pathway independent of the intestinal flora. The susceptibility of *Rig-I* ^−/−^ mice to induced CAC might be due to synergistic action of gut microbiota and inactivation of Rig-I signaling. MicroRNAs play crucial roles in CRC from pathogenesis to therapy [59]. As an RNA receptor, Rig-I could also bind to a large number of endogenous RNAs [26]. It is possible that Rig-I could bind to miRNAs to regulate their splicing and maturation, further to regulate the functions of their target genes.

At present, Rig-I is widely studied as an RNA virus receptor. Only our collaborators reported that Rig-I was a tumor suppressor and mice lack of Rig-I were more susceptible to DEN-induced hepatocellular carcinoma [39]. We also found decreased expression of Rig-I in colorectal tumor samples from patients and induced CRC of mice. In summary, our results demonstrated that Rig-I deficiency could lead to intestinal flora disturbance and was prone to colorectal cancer in mice. But how Rig-I affect the occurrence of colorectal cancer and in which the role of the gut flora still need to be further studied.

## Conclusion

Collectively, our study uncovered a novel aspect of Rig-I in monitoring gut microbiota through regulating IgA and IL6-STAT3-dependent Reg3γ pathway. Besides, Rig-I loss could also promote CRC progression both in the presence and absence of intestinal bacteria. This provides novel insights on CRC progression and reveals the potential therapeutic strategy. However, whether the inhibition of Rig-I on CRC was dependent on its immune protective effect needs further investigations.

## Additional files

Additional file 1:
**Figure S1.** Scoring criteria of RIG-I immunohistochemical staining. **Figure S2.** Analytical procedures of high-throughput sequencing data. **Figure S3.** Induction of colorectal tumors in mice. **a** The induction procedure of colorectal tumor. Wt mice (*n* = 10) and *Rig-I* ^−/−^ littermates (*n* = 11) were treated with AOM and DSS. All mice were sacrificed at the end of the procedure. **b** Colon length and diameter of wt and *Rig-I* ^−/−^ mice were shown. **c** H & E staining of untreated colon sections related to Fig 2c. Scale bar, 100 μm. **Figure S4.** Diversity of the gut microbiota between wild-type and *Rig-I* ^−/−^ mice. **a** *P*-values of P-tests on the NJ tree. The letter “W” and numbers represented week number. **b** Principal Component Analysis (PCA) was used to compare bacterial families across different groups. The percentage of variation explained by each principal component was indicated on the axis. **Figure S5.** Western blot analysis. **a** Western blot analysis in untreated mouse colons. **b** Western blot analysis in AOM/DSS-treated adjacent or tumor colons. **Table S1.** Primers used in this study. **Table S2.** Numbers of cases for each given score related to Fig. 2d and e. (DOCX 1089 kb)

## Abbreviations

Abx: Cocktail of antibiotics
AOM: Azoxymethane
CAC: Colitis-associated colorectal cancer
CRC: Colorectal cancer
DSS: Dextran sodium sulfate
IBD: Inflammatory bowel disease
PPs: Peyer’s patches
Rig-I: Retinoic acid-inducible gene-I
