# Supplementary material for: RNA virus receptor Rig-I monitors gut microbiota and inhibits colitis-associated colorectal cancer
Source: J Exp Clin Cancer Res. 2017 Jan 5;36:2. doi: 10.1186/s13046-016-0471-3 (PMC5217425; doi:10.1186/s13046-016-0471-3)
Supplement: Additional file 1: — Figure S1. Scoring criteria of RIG-I immunohistochemical staining. Figure S2. Analytical procedures of high-throughput sequencing data. Figure S3. Induction of colorectal tumors in mice. a The induction procedure of colorectal tumor. Wt mice (n = 10) and Rig-I −/− littermates (n = 11) were treated with AOM and DSS. All mice were sacrificed at the end of the procedure. b Colon length and diameter of wt and Rig-I −/− mice were shown. c H & E staining of untreated colon sections related to Fig 2c. Scale bar, 100 μm. Figure S4. Diversity of the gut microbiota between wild-type and Rig-I −/− mice. a P-values of P-tests on the NJ tree. The letter “W” and numbers represented week number. b Principal Component Analysis (PCA) was used to compare bacterial families across different groups. The percentage of variation explained by each principal component was indicated on the axis. Figure S5. Western blot analysis. a Western blot analysis in untreated mouse colons. b Western blot analysis in AOM/DSS-treated adjacent or tumor colons. Table S1. Primers used in this study. Table S2. Numbers of cases for each given score related to Fig. 2d and e. (DOCX 1089 kb) [file 13046_2016_471_MOESM1_ESM.docx]

**Additional file 1: Figure S1**


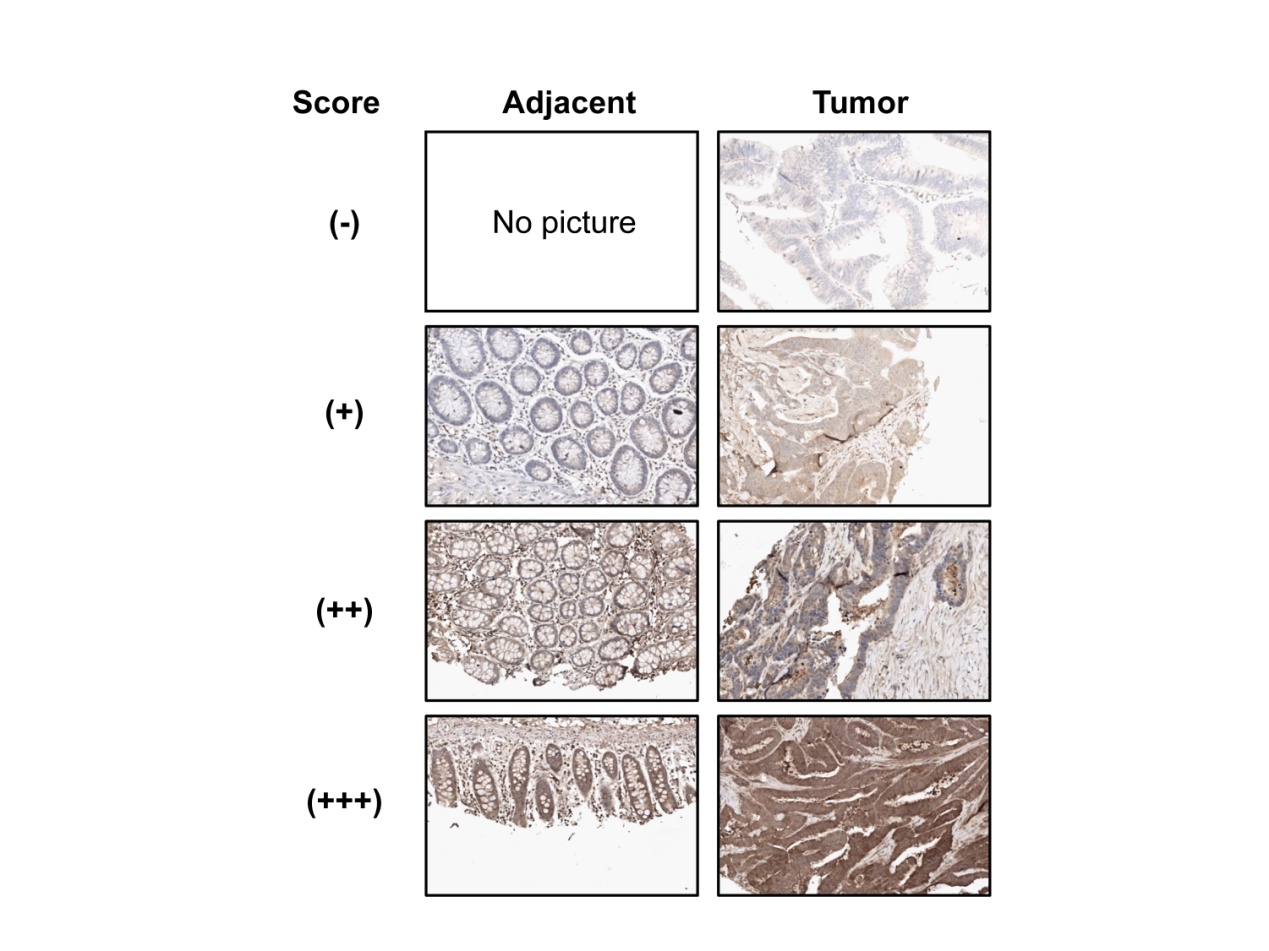


**Figure S2**


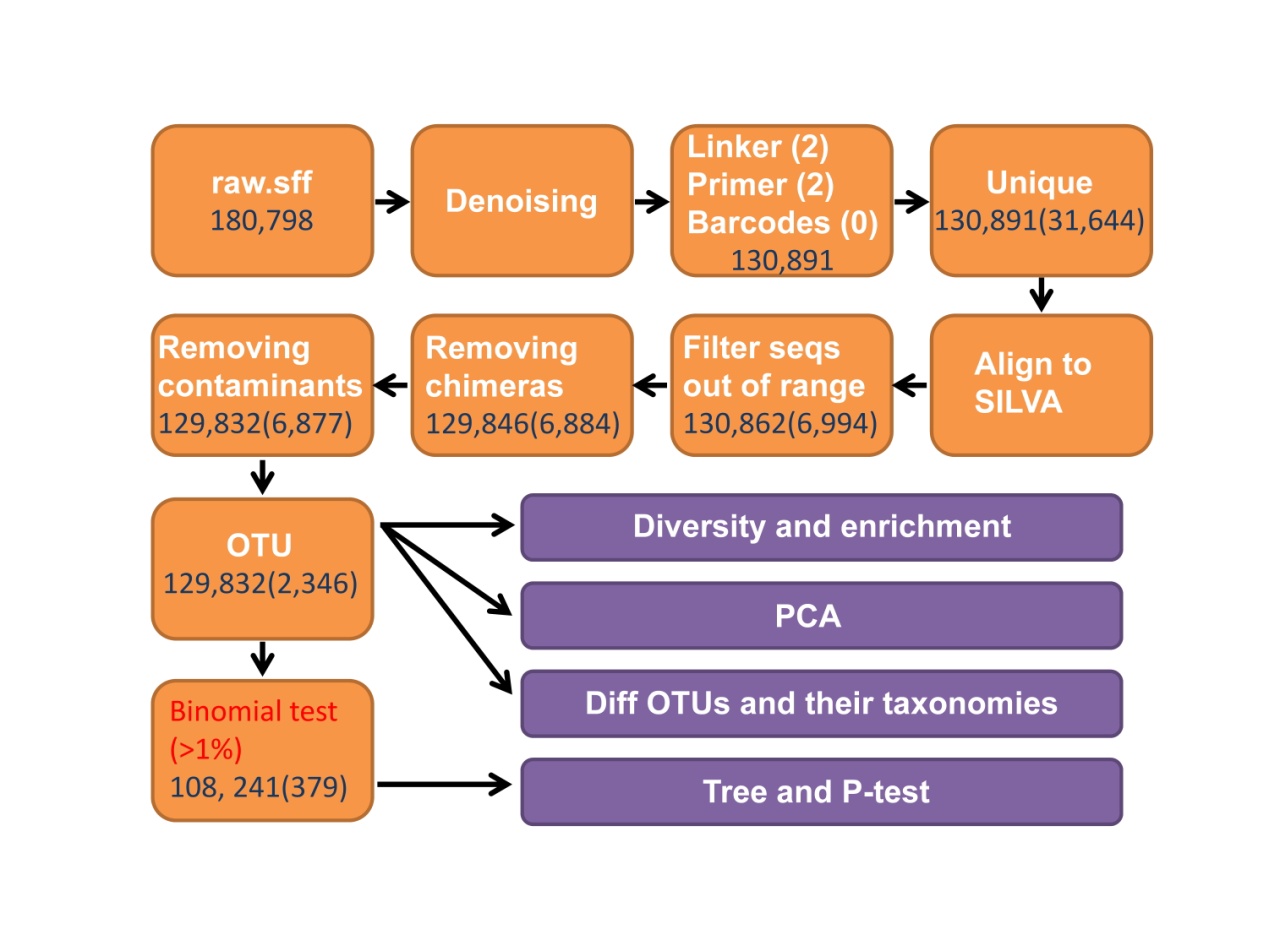


**Figure S3**


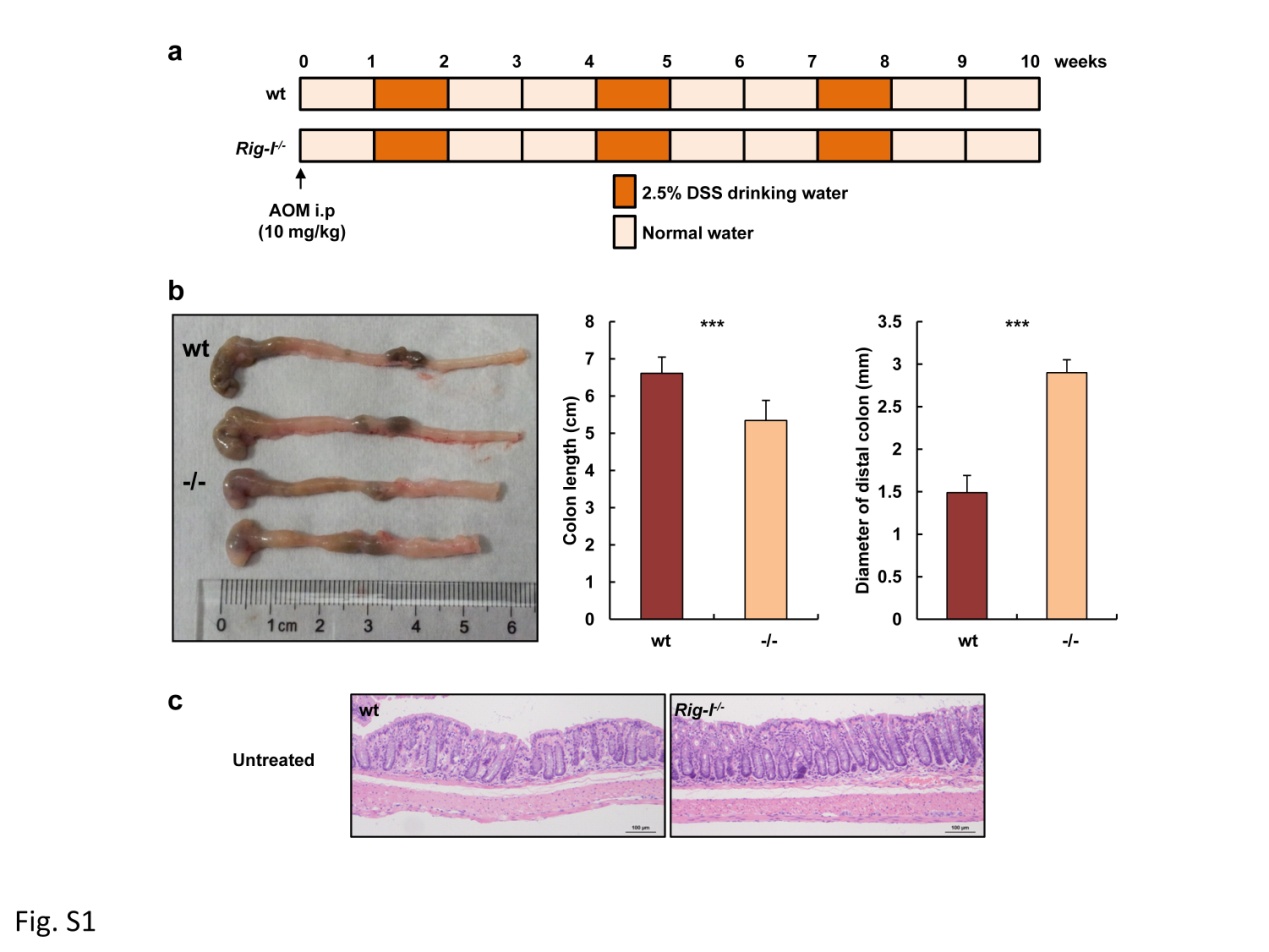


**Figure S4**


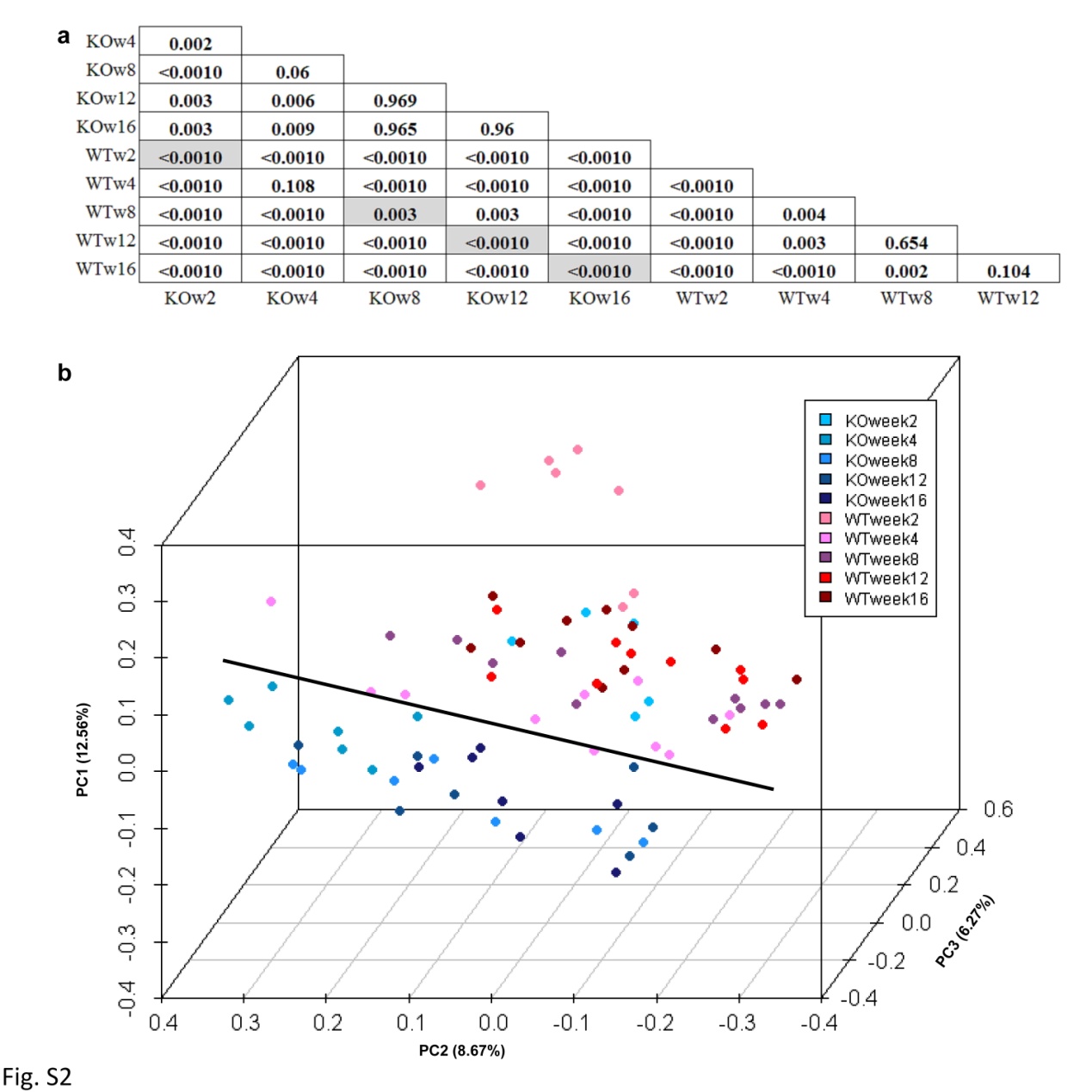


**Figure S5**


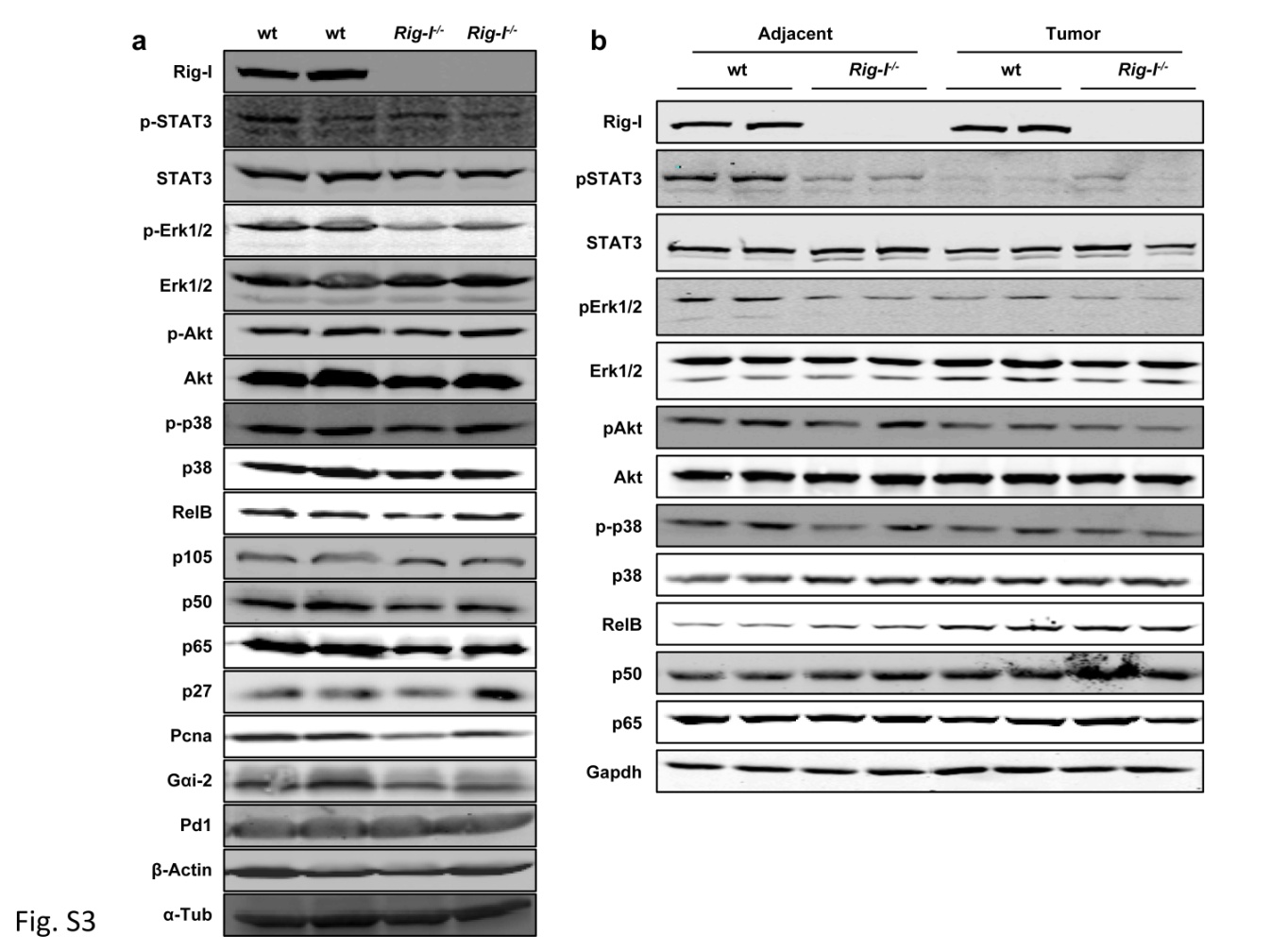


**Table S1. Primers used in this study.**

| Pd-1 (Pdcd1) | ACCCTGGTCATTCACTTGGG |
| --- | --- |
|  | CATTTGCTCCCTCTGACACTG |
| Cryptdin-1 | CTAGTCCTACTCTTTGCCCT |
|  | TTGCAGCCTCTTGATCTACA |
| Cryptdin-3 | GCTAGGGAGCACTTGTTTGC |
|  | TTGTTTGAGGAAAGGAGGCA |
| Cryptdin-4 | GTCCAGGCTGATCCTATCCA |
|  | GGGGCAGCAGTACAAAAATC |
| Cryptdin-5 | GTCCAGGCTGATCCTATCCA |
|  | GCCCAGGCTGATCCTATCCA |
| Il1b | CAACCAACAAGTGATATTCTCCATG |
|  | GATCCACACTCTCCAGCTGCA |
| Il6 | CAGGATACCACTCCCAACAGACC |
|  | AAGTGCATCATCGTTGTTCATACA |
| Il11 | TGTTCTCCTAACCCGATCCCT |
|  | CAGGAAGCTGCAAAGATCCCA |
| Tnfα | CATCTTCTCAAAATTCGAGTGACAA |
|  | TGGGAGTAGACAAGGTACAACCC |
| S100a9 | GCACAGTTGGCAACCTTTATG |
|  | TGATTGTCCTGGTTTGTGTCC |
| Gαi-2 | GCGCCTATGACTTGGTGCT |
|  | CTGCCTCGTCGTACTTGTTGG |
| Reg3γ | TTCCTGTCCTCCATGATCAAAA |
|  | CATCCACCTCTGTTGGGTTCA |
| Cyclin D1 | GCGTACCCTGACACCAATCTC |
|  | CTCCTCTTCGCACTTCTGCTC |
| Bcl-xl | GACAAGGAGATGCAGGTATTGG |
|  | TCCCGTAGAGATCCACAAAAGT |
| β-Actin | GGCTGTATTCCCCTCCATCG |
|  | CCAGTTGGTAACAATGCCATGT |

**Table S2. Numbers of cases for each given score related to Figs 2d and 2e.**

|  | Score | WT Count | KO Count |
| --- | --- | --- | --- |
| Inflammation | 1 | 2 | 0 |
|  | 1.5 | 4 | 0 |
|  | 2 | 3 | 0 |
|  | 2.5 | 0 | 0 |
|  | 3 | 0 | 4 |
|  | 3.5 | 0 | 1 |
|  | 4 | 0 | 2 |
|  | Total | 9 | 7 |
| Dysplasia | 0 | 2 | 0 |
|  | 0.5 | 2 | 0 |
|  | 1 | 1 | 0 |
|  | 1.5 | 1 | 0 |
|  | 2 | 1 | 0 |
|  | 2.5 | 0 | 1 |
|  | 3 | 2 | 1 |
|  | 3.5 | 0 | 4 |
|  | 4 | 0 | 1 |
|  | Total | 9 | 7 |
